# The diagnostic utility of multiphase CT (4D CT) in localizing parathyroid adenomas following negative or discordant 99mTc-sestamibi SPECT/CT and ultrasound imaging

**DOI:** 10.1093/bjr/tqag184

**Published:** 2026-08-12

**Authors:** Jake E Cowen, Xavier Fung, Matthew Ward, Hani Nasef, Darryl Meeking, David N Poller, Jasper Bekker

**Affiliations:** Department of Radiology, Queen Alexandra Hospital, Portsmouth Hospitals University NHS Trust, Portsmouth PO6 3LY, United Kingdom; Department of Radiology, Queen Alexandra Hospital, Portsmouth Hospitals University NHS Trust, Portsmouth PO6 3LY, United Kingdom; Department of Ear, Nose & Throat Surgery, Queen Alexandra Hospital, Portsmouth Hospitals University NHS Trust, Portsmouth PO6 3LY, United Kingdom; Department of Ear, Nose & Throat Surgery, Queen Alexandra Hospital, Portsmouth Hospitals University NHS Trust, Portsmouth PO6 3LY, United Kingdom; Department of Endocrinology, Queen Alexandra Hospital, Portsmouth Hospitals University NHS Trust, Portsmouth PO6 3LY, United Kingdom; Department of Pathology, Queen Alexandra Hospital, Portsmouth Hospitals University NHS Trust, Portsmouth PO6 3LY, United Kingdom; Department of Radiology, Queen Alexandra Hospital, Portsmouth Hospitals University NHS Trust, Portsmouth PO6 3LY, United Kingdom

**Keywords:** parathyroid adenoma, 4D CT, sestamibi, parathyroid gland, parathyroidectomy, SPECT/CT

## Abstract

**Objectives:**

To quantify the diagnostic utility of multiphase CT as a second-line imaging modality for parathyroid adenoma localization including assessing the differences between patients undergoing second-line imaging due to negative or discordant first-line imaging (MIBI SPECT/CT) results.

**Methods:**

Patients undergoing multiphase CT following negative or discordant first-line imaging were collected retrospectively over a 5-year period from 2019 to 2023. Seventy-three patients were included. Sensitivity, specificity, positive predictive value, negative predictive value, accuracy, positive and negative likelihood ratios of multiphase CT for preoperative parathyroid adenoma lateralization (correct side) and localization (correct quadrant) were calculated. Results for the whole cohort, negative imaging cohort, and discordant imaging cohorts were compared.

**Results:**

Fifty-eight of the included patients were female, and 15 were male. Mean age was 56.2 ± 11.8. One-hundred eighty-three quadrants and 98 sides were explored surgically. Thirty-seven patients (69.8%) underwent a minimally invasive surgical procedure. Across the whole cohort, multiphase CT demonstrated a sensitivity of 70.4% (95% CI: 58-81) and specificity of 86.4% (79-92). For lateralization, performance was higher with sensitivity 82.6% (72-91) and specificity 86.2% (68-96). Moderate positive likelihood ratios were also recorded as 5.26 (localization) and 5.99 (lateralization).

**Conclusions:**

Multiphase CT provided reliable diagnostic accuracy for localization and lateralization of parathyroid adenomas in the context of discordant or negative first-line imaging. Its high specificity and moderate positive likelihood ratios make it a valuable rule-in tool that enables targeted surgical approaches.

**Advances in knowledge:**

This study provides one of the largest datasets in the literature. Positive results suggest that CT can be considered in cases of negative or discordant first-line imaging if minimally invasive surgical techniques are desired.

## Introduction

Primary hyperparathyroidism (P-HPT) is the third most common endocrine disorder (behind diabetes and thyroid disease) with an estimated prevalence of 1-4 per 1000 individuals, and more common in female patients.^1^^,^ ^2^ Approximately 85% of primary hyperparathyroidism cases are caused by excessive PTH secretion by an adenoma of 1 of more of the parathyroid glands, resulting in hypercalcemia.^2^

Historically, nuclear medicine (NM) imaging has been the first line for detection and localization of parathyroid adenomas using the radioactive isotope technetium 99 m (99mTc) bound to 6 methoxyisobutylisonitrile (MIBI) ligands with fused images acquired from single-photon emission computed tomography (SPECT) and standard computed tomography (CT) in order to provide 3D localization.^3^ The diagnostic work up typically combines nuclear medicine imaging with a focused neck ultrasound (US) to add further anatomical information. In the last decade, multiphase CT (non-contrast, contrast enhanced-arterial phase, and contrast enhanced-delayed phase) imaging has emerged as a promising imaging technique with potentially improved sensitivity and specificity in the detection of parathyroid adenomas in comparison to Tc-MIBI SPECT/CT.^3^^,^ ^4^ Recent advances have also been made in NM with the development of F18-choline PET/CT (FCH-PET/CT), which has demonstrated encouraging results and has replaced MIBI SPECT/CT as first-line across Europe in nuclear medicine centers where FCH-PET is available.^5–7^ However, it is not currently widely adopted as first-line in the United Kingdom beyond specialist centers.

The Royal College of Radiologists iRefer guideline currently recommends the multimodality approach of ultrasound + multiphase CT or sestamibi as appropriate imaging in the localization of parathyroid adenomas.^8^ However, current NICE guidance is yet to reflect the benefits of CT or novel techniques such as FCH-PET and advocates for preoperative first-line imaging in the form of ultrasound and second line “usually sestamibi.”^9^ Further, it does not recommend further pre-operative imaging if ultrasound and sestamibi did not identify an adenoma. Nonetheless, in lieu of promising results in the literature, multiphase CT is being increasingly utilized in cases of non-localization with an awareness of the importance that accurate localization has on the avoidance of more invasive surgery.^10^

P-HPT secondary to parathyroid adenoma is curative by surgical resection with high success rates exceeding 95%.^11^ Traditional surgical approaches via bilateral 4-gland exploration have been refined in recent years with the development of focused, unilateral approaches with equal success and reduced complication rates, highlighting the importance of the accuracy of diagnostic imaging to correctly identify and localize the parathyroid adenoma ahead of surgery.^12^^,^ ^13^

This study aims to quantify the diagnostic utility of multiphase CT as a second-line imaging modality over a 5-year period at a large thyroid/parathyroid surgical center, capture any differences between patients undergoing second-line imaging due to negative or discordant first-line imaging results, and the potential value of second-line imaging on rationalizing downstream surgical approaches.

## Methods

### Patient selection

A retrospective review of the radiology information system (RIS) at Queen Alexandra Hospital, Portsmouth, UK was performed for patients with primary hyperparathyroidism undergoing multiphase contrast-enhanced CT (4D CT) neck imaging for localization of parathyroid adenomas following negative or discordant dual-tracer Tc-MIBI SPECT/CT and US between January 2019 and December 2023. The negative cohort was defined as patients with no adenoma localized on either US or NM studies. The discordant cohort included 1 negative study, contradictory or equivocal results which included low suspicion for a lesion. Patients with adenomas confidently identified on Tc-MIBI SPECT/CT did not proceed to second-line imaging.

Inclusion criteria included biochemical diagnosis of primary hyperparathyroidism (PTH > 6.4 pmol/L), negative or discordant results from first-line imaging and subsequent surgical exploration. Exclusion criteria included not proceeding to surgery, failure to undergo all 3 imaging studies, incorrect protocol (eg, single-phase CT-neck), previous neck surgery (including prior parathyroidectomy), and alternative histopathological diagnosis (eg, parathyroid carcinoma or multi-gland hyperplasia). Multi-gland hyperplasia was considered the diagnosis in patients with multiple enlarged parathyroid glands at time of surgery and with histopathology that supported a multi-gland hyperplasia, rather than multi-gland adenoma.

Imaging quadrants were split vertically by the midline and horizontally by mid-thyroid. The superior compartment extended superiorly from mid-thyroid to the angle of mandible. The inferior compartment extended inferiorly from mid-thyroid gland to the mediastinum. All Tc-MIBI SPECT/CT studies were reported by a single consultant in nuclear medicine. All CT were reported by consultant head & neck radiologists with access to the Tc-MIBI SPECT/CT and US result. Diagnostic data were extracted from radiology reports retrospectively. Scans were not re-reported or addended.

### Image acquisition

Parathyroid scintigraphy was performed using a dual-tracer subtraction protocol, comprising intravenous administration of Tc-99m pertechnetate (50 MBq) followed by Tc-99m sestamibi (850 MBq). Planar thyroid imaging was acquired 20 minutes after pertechnetate administration, followed by early planar sestamibi imaging at 10 minutes and SPECT/CT, with subtraction of the thyroid image from the sestamibi image to facilitate localization of hyperfunctioning parathyroid tissue.

Multiphase CT image acquisition was obtained with Canon 160 or 640-slice CT scanner. Patients were positioned headfirst, supine, and arms by their side. Topogram was performed from the supraorbital margin to the carina. A 3-phase acquisition protocol was used for all patients: an initial non-contrast scan from the hyoid to the head of the clavicles, followed by arterial and delayed phase acquisitions from the angle of mandible to the carina. The arterial phase was performed at 30 seconds and delayed phase at 80 seconds following injection of 75 mL of iodinated intravenous contrast at a rate of 4 mL/s. All scans were performed at 120 kV tube voltage. Slice thickness and collimation were 0.5 × 80, 0.5 × 64, and 0.5 × 64 for the non-contrast, arterial, and delayed phases, respectively.

### Data analysis

A Shapiro-Wilk test was performed for continuous variables and analyzed in combination with Q-Q plots to determine the distribution. Mean and standard deviation were calculated for normally distributed data and median and interquartile range for not normally distributed data.

Sensitivity, specificity, positive predictive value, negative predictive value, accuracy, positive and negative likelihood ratios of multiphase CT for preoperative adenoma lateralization (correct side) and localization (correct quadrant) were calculated with R (Version 2023.09.0 + 463). The 95% confidence intervals were calculated. Sensitivity and specificity were compared between discordant and negative first-line imaging cohorts using Fisher’s exact test due to small cell counts.

Ground truth for parathyroid adenoma was multiparametric assessment—imaging, surgical, and histopathological consensus. In cases of histopathological equivocation (eg, hyperplasia vs. adenoma), radiological and surgical correlation in combination with biochemical confirmation (intra-operative drop in PTH >50% and sustained biochemical improvement) were taken as evidence of adenoma resection.

Each CT provided 4 data points—1 for each quadrant (localization), or 2 data points—1 for each side (lateralization). True positive was interpreted as positive CT and positive histopathology. True negative was interpreted as negative CT and negative histopathology (eg, fatty tissue only, normal gland) (or negative surgical finding if normal gland identified and not removed). False positive was taken as positive CT with negative histopathology (or negative surgical finding if normal gland identified and not removed). False negative was interpreted as negative CT with positive surgical and histopathological consensus for adenoma.

### Ethical approval

Ethics approval was not deemed necessary because the study involved anonymous data from patient records only. The study did not involve human experiments or use of human tissue. Using the United Kingdom nationally available HRA research toolkit (https://www.hra-decisiontools.org.uk/ethics), this study was not deemed to require IRAS ethics. This service evaluation underwent institutional approval by the Portsmouth Hospitals University NHS Trust: Audit QIP ID 1009.

## Results

There were 73 patients that satisfied the inclusion criteria and underwent second-line multiphase CT for adenoma localization (Figure 1). Of these 73 patients, 54 were classified as having discordant first-line imaging, and 19 as having negative first-line imaging (Table 1 and Table 2).

**Figure 1:**
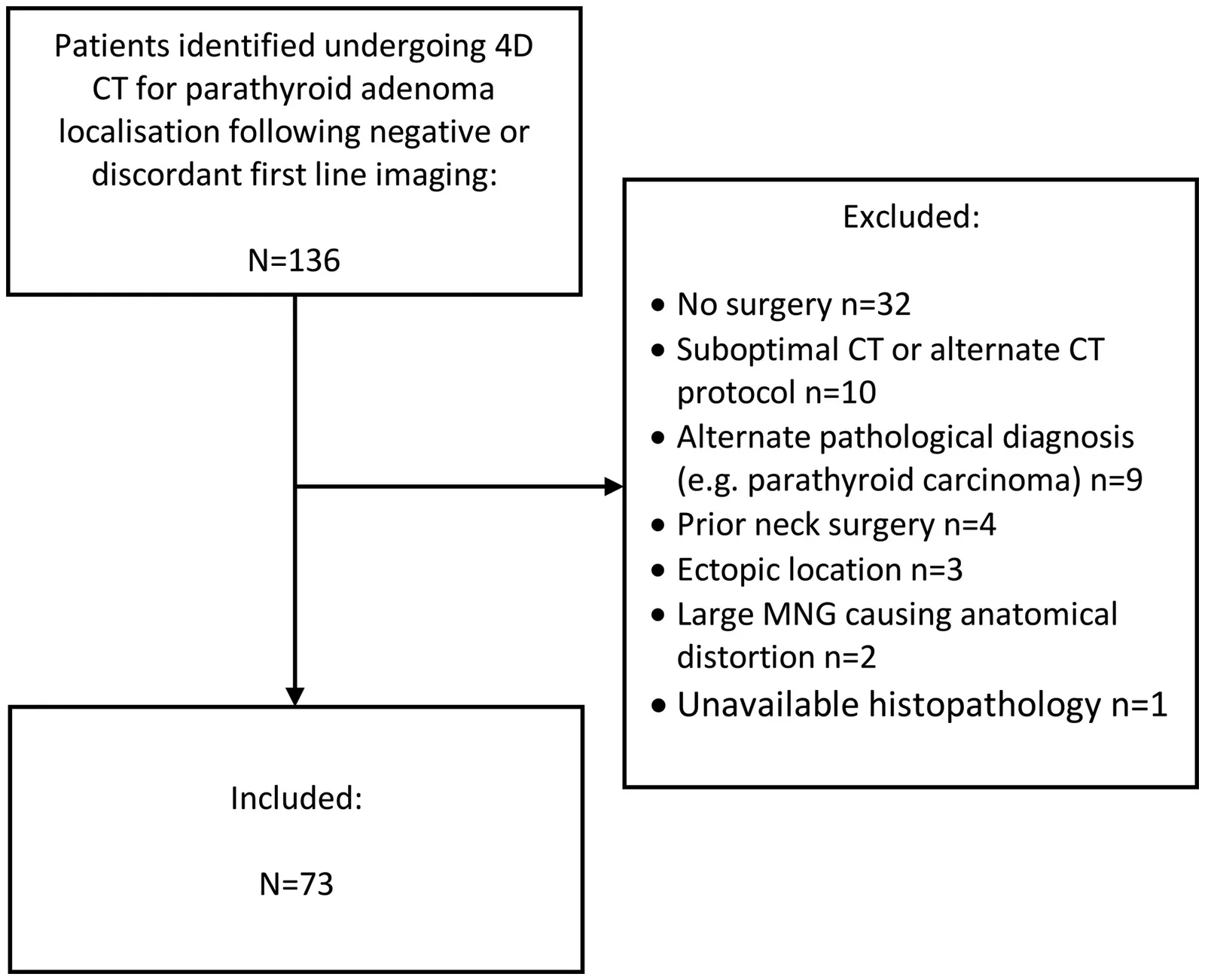
Flow chart of inclusion and exclusion data.

**Table 1:** Demographics.

Parameter	Value
Mean age, years ± SD	56.2 ± 11.8
Sex, *n* (%)	
Female	58 (79.5)
Male	15 (20.5)
Type of surgery, *n* (%)^a^	
Targeted single quadrant	5 (9.4)
Unilateral	32 (60.4)
Four quadrant exploration	16 (30.2)
Median pre-operative parathyroid hormone, pmol/L (IQR)	10.9 (9.0-14.0)
Median pre-operative corrected calcium, mmol/L (IQR)	2.75 (2.69-2.83)
Quadrants explored surgically, *n*	183
Sides explored surgically, *n*	98
Median lesion weight, g (IQR)^b^	0.61 (0.29-0.96)
Median lesion volume, cm^3^ (IQR)^c^	0.94 (0.60-1.73)

a n = 53 patients.

b *n* = 66 patients.

c *n* = 58 patients.

Fifty-eight of the included patients were female, and 15 were male. Mean age was 56.2 ± 11.8. Median pre-op PTH levels were 10.9 pmol/L (IQR 9.0-14.0) and median pre-op corrected calcium levels were 2.75 mmol/L (IQR 2.69-2.83) (Table 1).

In total, 183 quadrants and 98 sides were explored surgically. Nine patients underwent selective venous sampling (SVS) following multiphase CT (Figure 2). Excluding those that underwent SVS, and those who required concomitant surgery (eg, hemithyroidectomy), 37 (69.8%) patients underwent a minimally invasive surgical procedure (either unilateral or targeted single quadrant approach) as opposed to 16 (30.2%) undergoing 4 quadrant exploration. Histopathology demonstrated median gland weight for confirmed adenomas of 0.61 grams (IQR 0.29-0.96) with a median volume of 0.94 cm^3^ (IQR 0.60-1.73) (Table 1). No ectopic adenomas were detected in this cohort.

**Figure 2:**
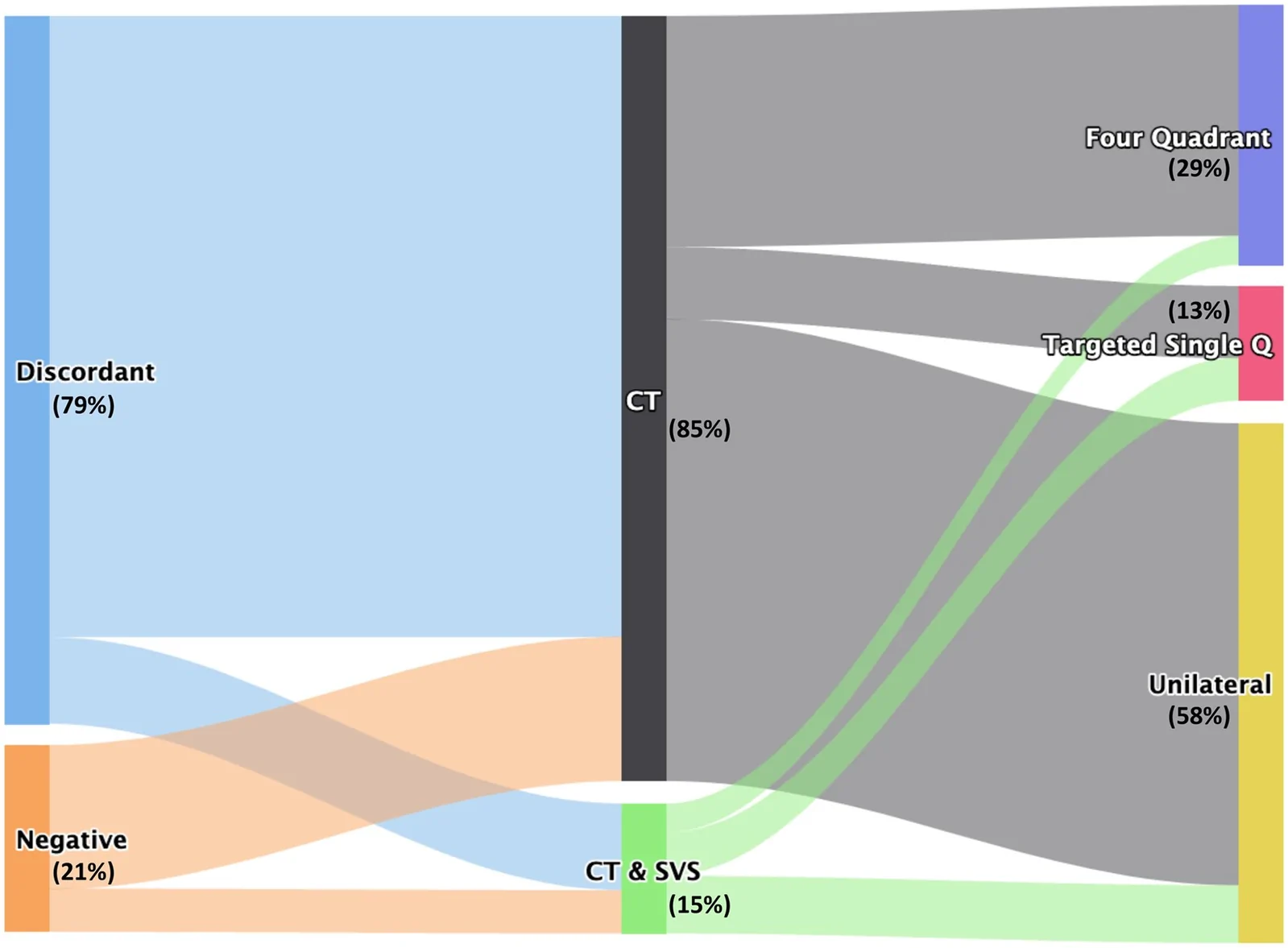
Sankey diagram demonstrating flow of patients through the service.

Across the whole cohort, multiphase CT demonstrated a sensitivity of 70.4% (95% CI: 58-81) and specificity of 86.4% (79-92) for localizing adenomas, with a corresponding positive predictive value (PPV) of 77.0% and negative predictive value (NPV) of 81.9%. For lateralization, performance was numerically higher with sensitivity 82.6% (72-91) and specificity 86.2% (68-96), giving an overall accuracy of 84.0% (75-90). The positive likelihood ratios (LR⁺) for localization and lateralization were 5.26 and 5.99, respectively, while the negative likelihood ratios (LR⁻) were 0.34 and 0.20 (Table 3) (Supplementary Material S2) (Figure 3).

**Figure 3:**
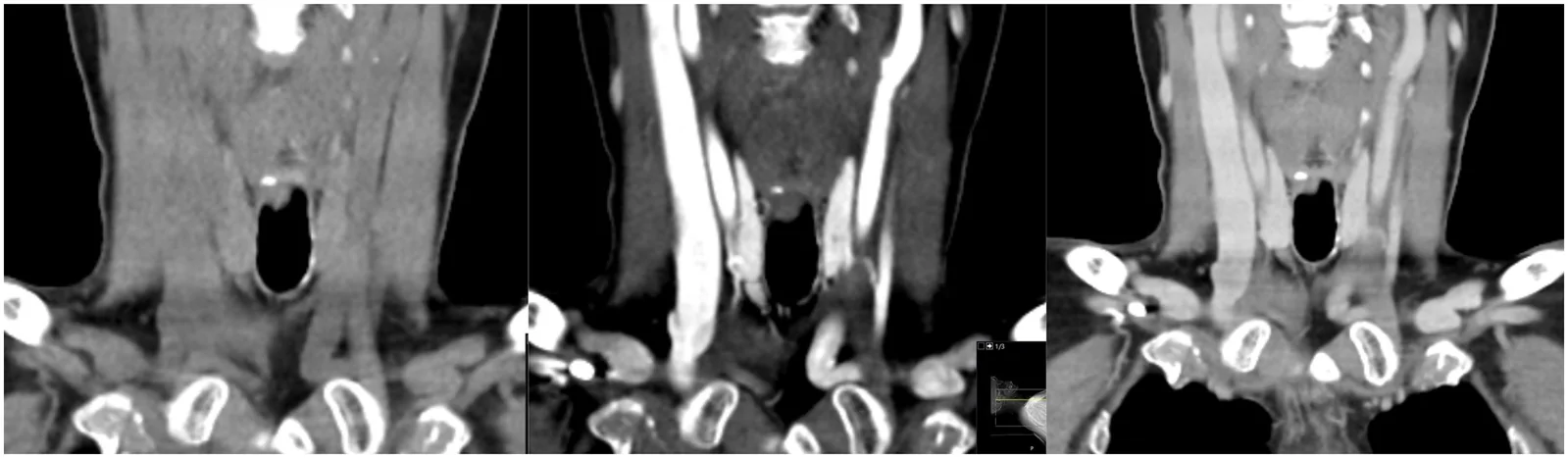
Parathyroid adenoma on multi-phase (4D) CT. Parathyroid adenoma seen inferior to the right lobe of the thyroid gland on non-contrast (left), arterial phase (middle), and delayed phase (right). The adenoma demonstrates less enhancement than the thyroid gland on arterial phase and delayed phase imaging, with greater washout seen (type B).

In the discordant first-line imaging cohort, sensitivity and specificity remained broadly comparable to the whole cohort (localization: 76.0% and 84.0%; lateralization: 88.0% and 85.7%) with accuracies of 80.9% and 87.3%, respectively. LR⁺ values were 4.74 for localization and 6.16 for lateralization, while LR⁻ values were 0.29 and 0.14 (Table 3).

In the negative first-line imaging cohort, CT retained useful diagnostic ability with sensitivities of 57.1% (34-78) and 68.4% (43-87), and notably high specificities of 93.6% (79-99) and 87.5% (47-100) for localization and lateralization, respectively (Table 3).

When comparing sensitivity and specificity between the discordant and negative first-line imaging cohorts, sensitivity for lateralization was numerically higher in the discordant cohort than the negative cohort (88.0% vs. 68.4%), although this did not reach statistical significance (*P* = .077). No significant differences were observed in localization sensitivity (*P* = 0.155) or specificity (*P* = .229) (Supplementary Material S3).

**Table 2:** First-line imaging results in the discordant and negative cohorts.

		Ultrasound			
		Correct/HS	Equivocal/LS	Incorrect	Not seen
**Tc-MIBI SPECT/CT**	**Correct/HS**		1	1	5
	**Equivocal/LS**	2	1	1	1
	**Incorrect**	0	1	1	4
	**Not seen**	29	3	4	19*

*Negative cohort.

Abbreviations: HS, high suspicion; LS, low suspicion.

**Table 3:** Sensitivity, specificity, PPV, NPV, accuracy, and likelihood ratios for each cohort by localization and lateralization (%, except LR).

	Whole cohort		Discordant first line		Negative first line	
	Localization	Lateralization	Localization	Lateralization	Localization	Lateralization
Sensitivity	70.4 (58-81)	82.6 (72-91)	76.0 (62-87)	88.0 (76-95)	57.1 (34-78)	68.4 (43-87)
Specificity	86.4 (79-92)	86.2 (68-96)	84.0 (74-91)	85.7 (64-97)	93.6 (79-99)	87.5 (47-100)
PPV	77.0 (65-86)	93.4 (84-98)	74.5 (60-86)	93.6 (82-99)	85.7 (57-98)	92.9 (66-100)
NPV	81.9 (74-89)	67.6 (50-82)	85.0 (75-92)	75.0 (53-90)	76.3 (60-89)	53.9 (25-81)
Accuracy	80.3 (74-86)	84.0 (75-90)	80.9 (73-87)	87.3 (77-94)	78.9 (65-89)	74.1 (54-89)
LR^+^	5.26 (3.21-8.62)	5.99 (2.40-14.97)	4.74 (2.81-7.98)	6.16 (2.15-17.65)	8.86 (2.20-35.58)	5.47 (0.85-35.11)
LR^-^	0.34 (0.24-0.49)	0.20 (0.12-0.34)	0.29 (0.17-0.47)	0.14 (0.06-0.30)	0.46 (0.28-0.76)	0.36 (0.18-0.74)

Abbreviations: LR−, negative likelihood ratio (95% confidence intervals); LR+, positive likelihood ratio; NPV, negative predictive value; PPV, positive predictive value.

PPV was high in both analyses (85.7%-92.9%), though NPV was more modest (53.9%-76.3%).

Accuracy was 78.9% for localization and 74.1% for lateralization. The LR⁺ values (8.86 and 5.47) indicate moderate rule-in potential, though the LR⁻ values (0.46 and 0.36) suggest weaker rule-out ability (Table 3).

## Discussion

This single-center analysis evaluates the use of multiphase CT as second-line imaging modality across a large tertiary thyroid/parathyroid center. The key findings are:

- Across all groups, CT demonstrated moderate to high diagnostic accuracy for both localization and lateralization of parathyroid adenomas, with higher sensitivity reported for lateralization.
- Compared with the discordant cohort, the negative first-line imaging group reported lower sensitivity for adenoma lateralization which did not reach statistical significance (*P* = .077) and no significant differences were observed in localization sensitivity or specificity.
- In ∼70% of the cohort, 4-quadrant exploration was avoided following second-line multiphase CT in favor of a more minimally invasive approach.

Since its inception in 2006, multiphase CT has been frequently utilized as either a first- or second-line imaging modality for the localization of parathyroid adenoma, with diagnostic performance considered greater than (or at least equal to) that of Tc-MIBI SPECT/CT.^14^^,^ ^15^ Specifically, as a second-line imaging modality, there have been fewer studies, and seldom those including >50 patients.

In the present study, multiphase CT demonstrated consistent and clinically meaningful diagnostic performance for both localization and lateralization of parathyroid adenomas across the whole cohort. Sensitivity ranged from 70% to 83%, with specificities of approximately 86% for both outcomes, translating to overall accuracies of 80% to 84%. PPVs were high (77%-93%), indicating that the vast majority of positive CT findings correctly predicted the presence of an adenoma confirmed at surgery. These results echo the limited literature base to date, including a recent meta-analysis suggesting a pooled sensitivity of 71% and PPV of 81%.^16–19^ Importantly, CT performance was comparable for localization and lateralization, with slightly higher sensitivity reported for side prediction, aligning with published evidence that lateralization is less technically demanding than precise quadrant localization.^16^^,^ ^18^^,^ ^20^^,^ ^21^

The NPV was more modest, particularly for lateralization (68%), reflecting that a negative scan did not reliably exclude disease. The positive likelihood ratios (LR⁺ ∼5-6) suggest that a positive CT finding makes the presence of an adenoma around 5× to 6× more likely, providing moderate evidence to “rule in” disease. Conversely, the negative likelihood ratios (LR⁻ ∼0.2-0.3) indicate only limited ability to “rule out” adenoma when CT is negative. Together, these metrics demonstrate that CT provides substantial diagnostic information for surgical planning and side localization, complementing the strengths and limitations of first-line ultrasound and Tc-MIBI SPECT/CT imaging.^14^^,^ ^22^^,^ ^23^

No studies to date have compared patients with discordant or equivocal first-line imaging with those having received both negative ultrasound and Tc-MIBI SPECT/CT. When performance was analyzed by first-line imaging status, CT performed in patients with discordant first-line studies maintained strong diagnostic performance (sensitivity 76%-88%, specificity 84%-86%), closely matching the results of the whole cohort. The high PPV (>93%) and LR⁺ values around 5-6 indicate that CT is particularly valuable for resolving inconclusive or conflicting ultrasound and Tc-MIBI SPECT/CT findings.

In contrast, the smaller negative first-line imaging cohort reported lower sensitivity (57%-68%) but notably higher specificity (88%-94%), yielding LR⁺ values up to 8.9. These comparisons were non-significant, although sensitivity of adenoma lateralization trended toward significance (*P* = .077), and in the context of a larger cohort may have yielded a significant result.

The high specificity observed in the whole cohort supports CT’s reliability in correctly identifying unaffected quadrants, helping to reduce unnecessary surgical exploration. Following second line, imaging in the present study, ∼70% of patients were able to undergo a minimally invasive surgical approach (either unilateral or targeted single quadrant) rather than 4-gland exploration. Avoidance of 4-gland exploration is favorable due to its potential risk of complications such as biochemical and symptomatic hypocalcemia, recurrent laryngeal nerve damage, and surgical scarring.^24^ However, while there is a theoretical risk, the existing comparative evidence, in the case of single adenomas located pre-operatively, has not yet reliably confirmed an increased risk in practice.^25^

Pre-operative imaging in primary hyperparathyroidism for parathyroid adenoma identification is an expanding field with a myriad of novel and advanced imaging techniques being utilized worldwide, including FCH-PET/CT and multiphase “4D” MRI.^26^^,^ ^27^ Over the past decade, FCH-PET has demonstrated promising results which have outperformed conventional Tc-MIBI SPECT/CT in a number of studies and at meta-analysis level, with sensitivity reaching as high as 98%.^27–31^ Greater sensitivity of first-line imaging may potentially reduce the need for second-line imaging altogether in this patient cohort. However, barriers to widespread implementation of FCH-PET/CT as first-line imaging in the United Kingdom include radiopharmaceutical availability, tracer cost, and access to PET facilities.^7^^,^ ^29^^,^ ^31^ With that said, evidence suggests that despite higher tracer costs with FCH-PET/CT, it’s use as first line where available is economically competitive owing to superior diagnostic performance, subsequent reduction in additional imaging and ability to facilitate minimally invasive parathyroidectomy.^32^

Pooled sensitivity for multiphase CT in first-line imaging has been reported as lower with a meta-analysis of 34 studies demonstrating a pooled sensitivity of 81% for lateralization and 73% for quadrant localization.^33^ Factors associated with discordance between preoperative multiphase CT and intraoperative findings include multi-gland disease, inferior position of the parathyroid lesion and lesions less than 10 mm.^34^ Direct comparison between FCH-PET/CT and multiphase CT in cases of negative or discordant first-line imaging have not been reported.

Consideration must also be given to the increased radiation dose in utilizing multiphase CT (or alternative second-line imaging techniques which involve ionizing radiation) as a second-line imaging modality, rather than proceeding straight to exploratory surgery following initial negative or discordant radiology. Multiphase CT has been reported to have higher radiation doses to the patient compared to sestamibi imaging with the dose to the thyroid reported as high as 57× higher with multiphase CT.^35^ Naturally, this carries a higher theoretical risk of malignancy in this cohort, especially in the context of additional imaging^36^ and risk-benefit discussions should be had with each patient. Performing CT using a dual-phase, rather than triple- or quadruple-phase imaging has been suggested as having similar sensitivity, specificity, and accuracy and allow for reduced radiation dose in this patient cohort.^37^ Alternatives to multiphase CT such as FCH-PET have had lower exposures reported in the literature, with doses of 6.8 mSv reported for MIBI SPECT/CT and as low as 2.8 mSv for FCH-PET/CT.^38^^,^ ^39^ Moreover, the added CT component for both hybrid imaging approaches was not felt to add significant radiation exposure (1.4 mSv for MIBI SPECT/CT, 0.8 mSv for FCH-PET/CT).^39^

This study is not without limitations. Subgroup analyses involved relatively small sample sizes, resulting in wide confidence intervals and reduced statistical precision for sensitivity, specificity, and likelihood ratio estimates. Moreover, the retrospective design introduces potential selection and verification bias, as not all patients may have undergone identical pathways (varying radiologists, surgeons and pathologists). Additionally, quadrant-level localization depends on intraoperative documentation and radiological interpretation, both of which may introduce classification variability. The cohort also reflects a single-center experience, limiting generalizability to centers with differing expertise, imaging protocols, or disease prevalence. Together, these factors indicate that while the observed diagnostic performance trends are robust, the estimates—particularly in smaller subgroups—should be interpreted with caution. In addition, a quadrant- and side-based analysis as our primary approach was adopted because it more directly reflects the clinical question of interest—namely, the ability of CT to accurately localize or lateralize parathyroid adenomas to guide surgical management. However, it is acknowledged that this approach assumes independence between observations and may therefore underestimate variability.

## Conclusion

Multiphase CT provided reliable diagnostic accuracy for localization and lateralization of parathyroid adenomas in the context of both discordant and negative first-line imaging (Tc-MIBI SPECT/CT + US). Its high specificity and strong positive likelihood ratios make it a valuable rule-in tool that enables targeted surgical approaches.

## Supplementary Material

tqag184_Supplementary_Data
