# Supplementary material for: The diagnostic utility of multiphase CT (4D CT) in localizing parathyroid adenomas following negative or discordant 99mTc-sestamibi SPECT/CT and ultrasound imaging
Source: Br J Radiol. 2026 Aug 12;99(1187):2398–405. doi: 10.1093/bjr/tqag184 (PMC13687754; doi:10.1093/bjr/tqag184)
Supplement: Supplementary file 1 [file tqag184_supplementary_data.docx]

Supplementary 1. Contingency tables for whole cohort, discordant cohort, and negative cohort.

| **Whole cohort localisation** | | Clinicopathological | | |
| --- | --- | --- | --- | --- |
|  |  | Positive | Negative | Total |
| 4D CT | Positive | 50 | 15 | 65 |
|  | Negative | 21 | 97 | 118 |
|  | Total | 71 | 110 | 183 |
| **Whole cohort lateralisation** | | Clinicopathological | | |
|  |  | Positive | Negative | Total |
| 4D CT | Positive | 57 | 4 | 61 |
|  | Negative | 12 | 25 | 37 |
|  | Total | 69 | 29 | 98 |
| **Discordant cohort localisation** | | Clinicopathological | | |
|  |  | Positive | Negative | Total |
| 4D CT | Positive | 38 | 13 | 51 |
|  | Negative | 12 | 68 | 80 |
|  | Total | 50 | 81 | 131 |
| **Discordant cohort lateralisation** | | Clinicopathological | | |
|  |  | Positive | Negative | Total |
| 4D CT | Positive | 44 | 3 | 47 |
|  | Negative | 6 | 18 | 24 |
|  | Total | 50 | 21 | 71 |
| **Negative cohort localisation** | | Clinicopathological | | |
|  |  | Positive | Negative | Total |
| 4D CT | Positive | 12 | 2 | 14 |
|  | Negative | 9 | 29 | 38 |
|  | Total | 21 | 31 | 52 |
| **Negative cohort lateralisation** | | Clinicopathological | | |
|  |  | Positive | Negative | Total |
| 4D CT | Positive | 13 | 1 | 14 |
|  | Negative | 6 | 7 | 13 |
|  | Total | 19 | 8 | 27 |

Supplementary 2. Likelihood ratio interpretation.

| **Likelihood ratio** | **Interpretation** |
| --- | --- |
| >10 | Strong evidence to rule in the pathology |
| 5-10 | Moderate evidence to rule in the pathology |
| 2-5 | Weak evidence to rule in the pathology |
| 0.5-2 | No evidence to rule in or rule out the pathology |
| 0.2-0.5 | Weak evidence to rule out the pathology |
| 0.1-0.2 | Moderate evidence to rule out the pathology |
| <0.1 | Strong evidence to rule out the pathology |

Supplementary 3. Significance testing between discordant and negative first-line imaging cohorts.

| **Metric** | **Comparison** | **Test** | **p** |
| --- | --- | --- | --- |
| Sensitivity | Localisation | Fisher’s exact | 0.155 |
| Sensitivity | Lateralisation | Fisher’s exact | 0.077 |
| Specificity | Localisation | Fisher’s exact | 0.229 |
| Specificity | Lateralisation | Fisher’s exact | 1.00 |
